# NEW EVIDENCE OF HEALTHIER AGING. POSITIVE COHORT EFFECT ON COGNITIVE DECLINE

**DOI:** 10.1093/geroni/igac059.2953

**Published:** 2022-12-20

**Authors:** Fernando Massa, Alejandra Marroig, Graciela Muniz Terrera, Scott Hofer

**Affiliations:** Universidad de la República, Montevideo, Montevideo, Uruguay; Universidad de la Republica Uruguay, Montevideo, Montevideo, Uruguay; Ohio University, Athens, Ohio, United States; University of Victoria, Victoria, British Columbia, Canada

## Abstract

Cross sectional studies have shown cohort effects in cognition, limited research exists about cohort effects on cognitive trajectories. Indeed, most longitudinal research conducted to study aging-related cognitive change focus on the association between risk factors and mean change in cognition, considering individual differences too, but longitudinal norms of cognitive function are less studied. In this study, we aim to test whether cohort effects exist across the distribution of verbal fluency trajectories, that is, whether cohort effects vary across different trajectory quantiles. With this purpose, we estimated norms using data from 9 waves of the English Longitudinal Study of Aging (ELSA). We considered the individuals born in the 1920s, 1930s, and 1940s to assess cohort effects. The methodological framework consisted of quantile mixed models where the effect of age was adjusted using splines. To test for possible cohort effects across the 5th, 50th and 90th quartiles, the coefficients associated with the splines varied among cohorts. Our results suggest that cognitive decline is less pronounced for individuals born in more recent decades (p < 0.001), supporting our hypothesis of cohort effects. Moreover, these results are consistent across quantiles (p-value < 0.001). Additionally, we found that quantiles of verbal fluency at a certain age is higher in participants from more recent cohorts compared to those in older cohorts. Our findings contribute to a better understanding of cognitive decline in older adults, demonstrating population changes over time at different levels of changes in verbal fluency.

